# Partial Mediation Role of Self-Efficacy between Positive Social Interaction and Mental Health in Family Caregivers for Dementia Patients in Shanghai

**DOI:** 10.1371/journal.pone.0083326

**Published:** 2013-12-26

**Authors:** Shuying Zhang, Helen Edwards, Patsy Yates, Qihao Guo, Chunbo Li

**Affiliations:** 1 Tongji University School of Medicine, Shanghai, China; 2 Department of Neurology and Institute of Neurology, Huashan Hospital, State Key Laboratory of Medical Neurobiology, Shanghai Medical College, Fudan University, Shanghai, China; 3 Institute of Health and Biomedical Innovation, Queensland University of Technology, Brisbane, Queensland, Australia; 4 Department of Biological Psychiatry Shanghai Mental Health Center, Shanghai Institute of Mental Health, Shanghai Jiao Tong University School of Medicine, Shanghai, China; Federal University of Rio de Janeiro, Brazil

## Abstract

We explored the mediation effect of caregiver self-efficacy on the influences of behavioral and psychological symptoms (BPSD) of dementia care recipients (CRs) or family caregivers’ (CGs) social supports (informational, tangible and affectionate support and positive social interaction) on CGs’ mental health. We interviewed 196 CGs, using a battery of measures including demographic data of the dyads, CRs’ dementia-related impairments, and CGs’ social support, self-efficacy and the Medical Outcome Study (MOS) Short-Form (SF-36) Health Survey. Multiple regression analyses showed that gathering information on self-efficacy and managing CG distress self-efficacy were the partial mediators of the relationship between positive social interaction and CG mental health. Managing caregiving distress self-efficacy also partial mediated the impact of BPSD on CG mental health. We discuss implications of the results for improving mental health of the target population in mainland China.

## Introduction

Over the last two decades, the theory of self-efficacy [1] has stimulated increasing research on dementia care [2]–[8]. Self-efficacy refers to a person’s belief in his or her capability to accomplish a specific task when facing a variety of situations [1]. Increasing levels of self-efficacy reflect increasing degrees of task difficulty that an individual believes he or she could manage [1].

Past research has **found** that family caregivers of persons with dementia (CGs) demonstrate significantly lower levels of self-efficacy than those caring for relatives with non-dementia diseases [9]. Most of the studies have emphasized the correlations between CG self-efficacy and health-related outcomes, particularly mental health outcomes, such as anxiety and depressive symptoms [2], [4]. According to Bandura [1], circumstances (or external factors), such as task demands and support from others, are a key influence on self-efficacy. In the dementia care literature, impairment of care recipients (CRs) was the most difficult task CGs had to manage in the course of caregiving [10]–[14]. Of the impairments, behavioral and psychological symptoms of dementia (BPSD) [15] have been found the primary challenge impairing CGs' sense of self-efficacy and mental health [3], [6], [8], [16]. On the other hand, social support has been regarded as an external factor enhancing CGs’ belief in their capability for managing care [2], [3], [6], [17], [18] and for improving CG mental health [2], [3], [6], [17]. A significant and positive relationship has been found between social support and CG self-efficacy using a range of social support and self-efficacy measures [2], [3], [17], [18].

However, the relationships among caregiver self-efficacy, the two external factors (impairments of CRs and social support of CGs), and CGs’ mental health still need further clarification. Compared to the studies of the direct influences of the two external factors on caregiver self-efficacy and mental health, there is limited research exploring indirect influence, particularly the way by which caregiver self-efficacy influences relationships between the two external factors and CGs’ mental health [17]. A study [17] conducted in Hong Kong reported that caregiver self-efficacy partially mediated the relationship between social support and CGs’ depression symptoms. Many previous studies on the relationship between social support and caregiver self-efficacy measured one or two types of social support (such as emotional and practical support [18]) or scope of social network [2], and explored the associations of the social support with specific domains of CG self-efficacy [2], [3], [18]. For example, greater social support was associated with a stronger sense of self-efficacy with respect to obtaining respite and responding to disruptive behavior [17], or with respect to self-care self-efficacy and problem solving self-efficacy [3].

Our previous study used the Chinese versions of the Medical Outcome Study Social Support Survey (MOS-SSS) [19] and Self-Efficacy Questionnaire for Chinese Family Caregivers (SEQCFC) [16], [20]. We found, after adjusting for impairments of care recipients (CRs), significant associations of MOS-SSS total score with four domains of caregiver self-efficacy (gathering information, obtaining support, responding to BPSD, and managing caregiving distress) [16]. There were limited studies focusing on the mediating effects of caregiver self-efficacy on the influences of dementia-related impairments on CGs’ mental health. In addition, fewer studies exploring mediation effects of a specific domain of caregiver self-efficacy on the influences of main types of social support or on CGs’ mental health. Earlier, we reported inverse and significant associations between BPSD and three domains of caregiver self-efficacy (responding to BPSD, managing routine care, and managing caregiving distress) in Chinese CGs [16]. However, there is a paucity of research exploring mediating effects of specific domains of caregiver self-efficacy on the relationship of CRs’ impairments to CGs’ mental health. Moreover, in terms of the mediating role of caregiver self-efficacy, few empirical explorations have been reported in mainland China.

**Figure 1:**
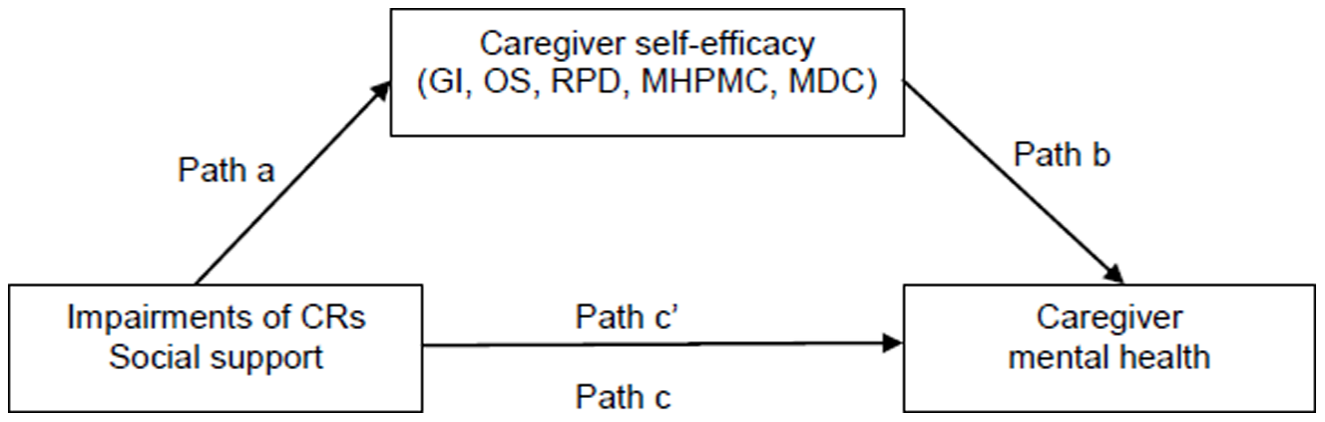
Hypothesized mediating effects of on the relationships between impairments of CRs, social support and caregiver mental health. Path a represents the influence of predictors (impairments of care recipients and caregiver social support) on the mediators (five domains of caregiver self-efficacy). Path b represents the influence of meidators on outcome measure (caregiver mental health). Path c represents the direct effects of predictors on outcome measure, and Path c’ demonstrates the predictors indirectly influence outcome measure via the influence of the mediators.

Therefore, adapted from Bandura’s self-efficacy theory [1] and the relevant caregiver research including our previous explorations [16], we used the Chinese versions of social support (MOS-SSS) [19] and caregiver self-efficacy measures (SEQCFC) [16] to further explore whether five domains of caregiver self-efficacy (gathering information, obtaining support, responding to BPSD, managing routine care and managing caregiving distress) mediate the relationships (a) between CRs’ impairments and CGs’ mental health, and (b) between four aspects of social support (informational, tangible and affectionate support and positive social interaction) and CGs’ mental health (Figure 1).

## Methods

### Participants

A cross-sectional study was designed. We studied a convenience sample of 196 CGs. We recruited CGs when they accompanied CRs to see neurologists at the neurological outpatient department of Shanghai Huashan Hospital. The recruitment and data collection procedures were reported in a previous article [16]. CG inclusion criteria were that: (a) the individual family CG provided the majority of caregiving for the CR, (b) the CG was over 18 years old, and (c) the CR was diagnosed dementia by a neurologist based on the DSM-IV. We excluded CGs who were below 18 years old or who simultaneously provided care for another relative with a chronic disease. Permission to use the standard instruments for this study was obtained from the original authors. Ethical approval to conduct the study was obtained by the designated hospital and the Human Research Ethics Committee of Queensland University of Technology. The participants were all voluntary, and written informed consent was obtained prior to the investigation. They were assured of their confidentiality and anonymity and informed that their decision to participate in or withdraw from the study would not impact on their current or future relationship with the hospital. The participants were also given the contact details of the hospital if they had any concerns or complaints about the ethical conduct of this study.

The mean age of CRs was 72.9 years (SD = 8.60). Most (53.1%) were 75 or older. Of 196 CRs, 101 had been diagnosed with very mild or mild dementia; 40 were at a moderate stage and 55 at a severe stage. The average age of CGs was 63.8 (SD = 12.85). Most (*n* = 168, 85.7%) were over 50, and the oldest CG was 90 years old. The majority of the CRs (n = 107; 54.6%) were female, as were the CGs (118; 60.2%). Most CGs (137; 69.9%) were spouses of the CR (79 wives, 58 husbands).

### Measures

**Caregiver mental health.** CG mental health was the dependent variable targeted in this study. The mental component summary score (MCS) of the Chinese version of the Medical Outcome Study (MOS) Short-Form (SF-36) Health Survey [21] was used to measure CGs’ mental health. The MCS was derived from subscale scores of SF-36. The SF-36 comprises eight subscales: physical functioning (PF), role limitations due to physical health problems (RP), bodily pain (BP); general health perceptions (GH), vitality (VT), social functioning (SF), role limitations due to emotional problems (RE) and mental health (MH). The Chinese version of SF-36 has been extensively used in diverse populations in mainland China and the psychometric properties have been found acceptable [21]. The Cronbach’s alpha coefficients ranged from .72 to .88 except the VT (.66) and SF subscale (.39). The two-week test-retest reliabilities ranged from .66 to .94. The convergent and discriminant validities were also acceptable [25].

**Dementia-related impairments.** The CRs’ Mini-Mental Status Examination (MMSE) scores were obtained from their medical records. CGs reported levels of CR disability using the Chinese version of the Disability Assessment in Dementia (DAD) scale [22]. The DAD measures the instrumental activities of daily living (DAD-IADLs, 25 items) and activities of daily living (DAD-ADLs, 22items) over the most recent two-week period. Each item is divided and assessed three main aspects of executive function: initiation, planning and organization, and effective performance. Each item is scored as “0” (cannot perform the activity without assistance or reminder), “1” (can perform the activity without assistance or reminder), or “non applicable” (not do or not having opportunity to do the activity). The total score of the scale or each subscale is yielded by adding the corresponding questions and converting the score from a range from 0 to 100. Higher scores indicate better physical functioning. The reliability and validity of DAD were satisfactory (Cronbach’s α for the total score, .91). Test-retest reliability and interrater reliability (intraclass correlation coefficients) were .99 and .98, respectively. Total DAD score correlated well with measures of global deterioration to that of global deterioration [22]. CGs also reported BPSD with the Chinese version of the 24-item Revised Memory and Behavior Problems Checklist (RMBPC) [23]. The scale measures three aspects of BPSD: memory-related problems, depression, and disruption problems. CGs rated BPSD on a 5-point scale from “0” (never occurs) to “4” (occurs daily or more often), yielding a total score ranging from 0 to 96. The Cronbach’s α for the total score was .816, and the test-retest reliability was .89 (*P*<.001) [23]. The RMBPC has been extensively applied to examine the cognitive, emotional, and functional impact of caregiving, and the results showed satisfactory convergent validity [23]–[25].

**Social support.** The Chinese version of the Medical Outcome Study Social Support Survey (MOS-SSS) [19] was used to measure the social support that the CGs perceived. The MOS-SSS assesses four types of social support: emotional and informational (8 items), tangible (4 items), affectionate (3 items), and positive social interaction (4 items). Each item is rated on a 5-point Likert from “1” (none of the time) to “5” (all of the time). The total score and score of each subscale are transformed to 0-100, with higher scores indicating more social support. The Cronbach’s alpha for the Chinese version of MOS-SSS and subscales were all over 0.80, and the results of concurrent validity test were satisfactory [19].

**Caregiver self-efficacy.** The Self-Efficacy Questionnaire for Chinese Family Caregivers (SEQCFC) [16], [20] was used to measure caregiver self-efficacy. The 27-item questionnaire assesses self-efficacy of Chinese CGs for five domains of caregiving activities: gathering information about treatment, symptoms and health care (GI subscale, 4 items); obtaining support (OS subscale, 6 items); responding to behavior disturbances (RBD subscale, 7 items); managing household, personal and medical care (MHPMC subscale, 4 items); and managing distress associated with caregiving (MDC subscale, 6 items). The total scale and subscale scores are rated from 0% (“cannot do at all”) to 100% (“certainly can do”), with higher score indicating stronger sense of caregiver self-efficacy. All Cronbach’s alpha coefficients were over .80. The four-week test-retest reliabilities ranged from .64 to .85. The results of convergent validity were also acceptable [20].

### Data Analysis

In a mediation model, predictors should significantly influence both outcome (Figure 1, Path c) and the mediator (Figure 1, Path a), and the mediator needs to significantly associate with the outcome (Figure 1, Path b) [26]. The multiple regression analyses reported here tested the following mediation effects (Figure 1): To test the primary predictors (IVs) of outcome (Figure 1, Path c), CGs’ mental health score (MCS) regressed on the dementia-related impairments (MMSE and RMBPC, DAD–ADLs and DAD-IADLs) and four social support variables (four subscales of MOS-SSS). To identify the predictors of mediator (Figure 1, Path a), five caregiver self-efficacy measures (subscales of SEQCFC) were employed as dependent variables (DVs), respectively. Each caregiver self-efficacy measure regressed on the dementia-related impairments and four social support variables. To test the significant effect of the mediator on outcome, the CGs’ MCS regressed on the five caregiver self-efficacy measures (Figure 1, Path b); and then (d) hierarchical multiple regression analysis was selected for mediation effect testing. The mediation effect found is that a reduced effect of the IV on MCS occurred when the mediator entered the equation (Figure 1, Path c’). Sobel tests were conducted to test the significance of the mediation effects [27]. Prior to the analysis, normality, linearity, homoscedasticity and absence of multicollinearity were tested and ensured. All analyses were conducted using SPSS 16.0.

## Results

Table 1 presents the means and SDs for the variables in the analyses. The results of regression of dementia-related impairments, social support on CGs’ mental health (Figure 1, Path c) are presented in Table 2. The compound influence of dementia-related impairments and four social support variables was significant on CGs’ mental health (as measured by MCS). The CRs’ BPSD (total score of RMBPC) and CGs’ score for positive social interaction were two predictors of CGs’ mental health (Figure 1, Path c).

**Table 1:** Descriptive statistics for MMSE, DAD, RMBPC, MOS-SSS, caregiver self-efficacy and mental health.

Variables			Mean	SD
Care recipient (n = 196)				
	MMSE		13.52	8.21
	DAD-ADLs (%)		72.64	29.82
	DAD-IADLs (%)		46.76	34.85
	RMBPC		27.98	14.20
Caregiver				
	Social support (MOS-SSS) (n = 196)			
		Emotion & Information	53.81	26.04
		Tangible support	58.86	30.91
		Affectionate support	34.82	26.89
		Positive social interaction	51.19	28.99
	Caregiver self-efficacy (SEQCFC)			
		Gathering information (n = 196)	57.92	24.75
		Obtaining support (n = 186)	69.03	27.25
		Responding to BPSD (n = 174)	65.66	21.29
		Managing routine care (n = 194)	82.81	17.11
		Managing distress (n = 190)	67.63	20.32
	Mental health (MCS) (n = 196)		45.22	10.88

**Abbreviations:** MMSE, Mini Mental Status Examination; DAD-ADLs, Activity of Daily Living Subscale of Disability Assessment in Dementia; DAD-ADLs, Instrumental Activities of Daily Living Subscale of Disability Assessment in Dementia; RMBPC, Revised Memory and Behaviour Problems Checklist; MOS-SSS, Medical Outcome Study Social Support Survey; SEQCFC, Self-Efficacy Questionnaire for Chinese Family Caregivers; MCS, Mental Component Summary score (MCS) of the Medical Outcome Study (MOS) Short-Form (SF-36) Health Survey.

**Table 2:** Regressions of dementia-related impairments, social support on caregiver mental health (path c).

			MCS		
Independent Variables			β	t	Sig.
Constant				9.355	.000
Impairments of care recipient					
	DAD-ADLs		.16	1.393	.165
	DAD-IADLs		–.15	–1.244	.215
	RMBPC		–.21	–2.749	.007
Social support (MOS-SSS)					
	Emotion & Information		–.01	–.109	.914
	Tangible support		.12	1.659	.099
	Affectionate support		–.02	–.212	.832
	Positive social interaction		.34	4.376	.000
***R^2^*(adj.)**		.19			
***F***		7.491***			

**Abbreviations:** MCS, Mental Component Summary score (MCS) of the Medical Outcome Study (MOS) Short-Form (SF-36) Health Survey; DAD-ADLs, Activity of Daily Living Subscale of Disability Assessment in Dementia; DAD-ADLs, Instrumental Activities of Daily Living Subscale of Disability Assessment in Dementia; RMBPC, Revised Memory and Behaviour Problems Checklist; MOS-SSS, Medical Outcome Study Social Support Survey.

* P≤.05; **P≤.01; ***P≤.001.

The results of the regression analyses for Path a (Figure 1) are presented in Table 3. The overall influence of dementia-related impairments and four types of social support was significant on each caregiver self-efficacy measure. For the predictors of each domain of caregiver self-efficacy, three social support variables, including informational, affectionate support and positive social interaction support, had a positive influence on CGs’ gathering information self-efficacy (GI). Positive social interaction support also had positive influence on CGs’ responding to BPSD (RBD) and managing caregiving distress (MDC) self-efficacy. Tangible support was positive associated with obtaining support (OS) and MDC self-efficacy. CRs’ BPSD (RMBPC) tended to weaken three aspects of caregiver self-efficacy (responding BPSD, managing routine care and managing caregiving distress self-efficacy); and CG's IADLs score (DAD-IADLs) was another impairment variable having negative impact on MDC self-efficacy.

**Table 3:** Regressions of dementia-related impairments and social support on caregiver self-efficacy (path a).

	Dependent Variables									
Independent Variables	GI		OS		RBD		MHPMC		MDC	
	β	t	β	t	β	t	β	t	β	t
Constant		4.657***		3.405***		7.099***		16.167***		7.731***
DAD-ADLs	–.04	–.297	.12	1.074	–.06	–.503	–.17	–1.392	.10	.844
DAD-IADLs	–.05	–.379	–.20	–1.597	–.06	–.473	–.09	–.674	–.25	–1.976*
RMBPC	–.03	–.377	–.13	–1.682	–.24	–2.839**	–.27	–3.355***	–.17	–2.127*
Emotion & Information	–.18	–2.108*	.07	.885	.07	.785	–.06	–.666	–.13	–1.529
Tangible support	.08	1.079	.27	3.576***	.05	.579	.01	.067	.21	2.741**
Affectionate support	.26	2.983**	.14	1.604	.03	.358	–.05	–.560	.03	.354
Positive social interaction	.23	2.886**	.11	1.371	.25	2.956**	.05	.626	.27	3.268***
***R^2^*(adj.)**	.11		.20		.12		.05		.13	
***F***	4.405***		7.419***		4.377***		2.532*		5.066***	

**Abbreviations:** GI, Self–Efficacy for Gathering Information about Treatment, Symptoms and Health Care; OS, Self-Efficacy for Obtaining Support; RBD, Self-Efficacy for Responding to Behavior Disturbances; MHPMC, Self-Efficacy for Managing Household, Personal and Medical Care; MDC, Self-Efficacy for Managing Distress Associated with Caregiving; DAD-ADLs, Activity of Daily Living Subscale of Disability Assessment in Dementia; DAD-ADLs, Instrumental Activities of Daily Living Subscale of Disability Assessment in Dementia; RMBPC, Revised Memory and Behaviour Problems Checklist.

* P≤.05; **P≤.01; ***P≤.001.

The results of regression analysis for Path b (Figure 1) are presented in Table 4. The compound influence of five caregiver self-efficacy measures was significant on MCS. Three predictors to CGs’ mental health were identified, including GI, MHPMC (managing routine care) and MDC self-efficacy. CGs reporting higher levels of GI, and MDC self-efficacy reported better mental health. Interestingly, CGs having stronger sense of MHPMC self-efficacy reported worse mental health.

**Table 4:** Regressions of caregiver self-efficacy on caregiver mental health (path b).

	MCS		
Independent Variables	β	t	Sig.
Constant		7.863	.000
GI	.22	2.794	.006
OS	.11	1.364	.174
RBD	.06	.650	.517
MHPMC	–.17	–2.198	.029
MDC	.37	4.448	.000
***R^2^*(adj.)**	.28		
***F***	13.677***		

**Abbreviations:** MCS, Mental Component Summary score (MCS) of the Medical Outcome Study (MOS) Short-Form (SF-36) Health Survey; GI, Self-Efficacy for Gathering Information about Treatment, Symptoms and Health Care; OS, Self-Efficacy for Obtaining Support; RBD, Self-Efficacy for Responding to Behavior Disturbances; MHPMC, Self-Efficacy for Managing Household, Personal and Medical Care; MDC, Self-Efficacy for Managing Distress Associated with Caregiving.

* P≤.05; **P≤.01; ***P≤.001.

From the results of analysis for Path b, two self-efficacy measures (OS and RBD self-efficacy) which had insignificant influences on outcome measure were not included as the second group of IVs in the corresponding regression equations for the mediation testing. Therefore, three hierarchical multiple regression equations were conducted to test the mediation effects of three domains of caregiver self-efficacy (GI, MHPMC and MDC), respectively. To test the mediation role of GI self-efficacy, CGs’ score for positive social interaction support entered as the first group of IVs, as it was the predictor for both the potential mediator and the outcome measure. Similarly, CRs’ score for RMBPC entered as the first group of IVs to test the mediation role of MHPMC self-efficacy; and to test the mediation role of MDC self-efficacy, both positive social interaction support and RMBPC entered as the first group of IVs.

**Figure 2:**
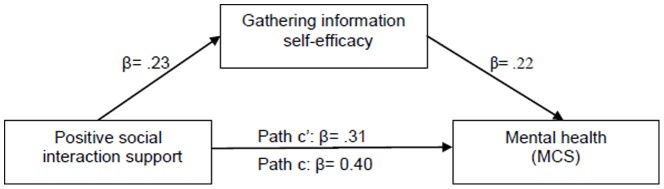
Partial mediating effect of gathering information self-efficacy on the relationship between positive social interaction and caregiver mental health.

**Figure 3:**
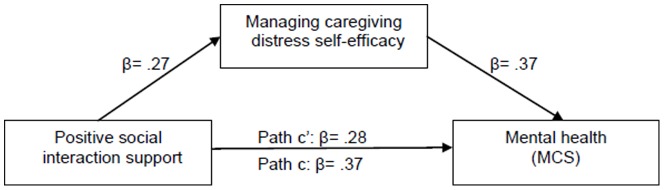
Partial mediating effect of managing caregiving distress self-efficacy on the relationship between positive social interaction and caregiver mental health.

**Figure 4:**
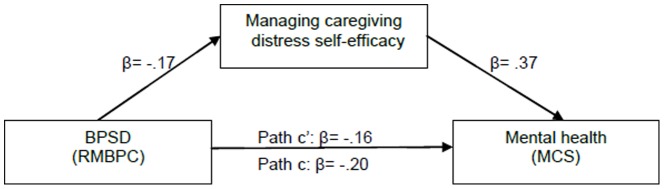
Partial mediating effect of managing caregiving distress self-efficacy on the relationship between BPSD and caregiver mental health.

The composite influences of the IVs in the corresponding hierarchical multiple regression equations were significant on CGs’ mental health, respectively [GI: *F* (2,195)  = 32.161, *P*<.001; MHPMC: *F* (2,193)  = 6.460, *P* = .002; MDC: *F* (2,189)  = 27.323, *P*<.001]. After adjusting for the influence of positive social interaction scores, GI self-efficacy accounted for significant portions of mental health variance (GI: β = .32, △R^2^ = .09, *P*<.001), and the β values for positive social interaction were reduced from .40 (*P*<.001) to .31 (*P*<.001) when GI self-efficacy entered. After adjusting for the influence of positive social interaction support and RMBPC, MDC self-efficacy accounted for significant portions of mental health variance (MDC: β = .35, △R^2^ = .11, *P*<.001). When MDC self-efficacy entered, the β values for positive social interaction were reduced from .37 (*P*<.001) to .28 (*P*<.001), and the β values of RMBPC were also increased from –.20 (*P* = .003) to –.16 (*P* = .009). From the results of Sobel tests, GI self-efficacy (2.359, Std. Error  =  0.13, *P* = 0.02; see Figure 2) and MDC self-efficacy (3.119, Std. Error  =  0.20, *P* = 0.001; see Figure 3) partially mediated the relationship between positive social interaction and CGs’ mental health. MDC self-efficacy was also the partial mediator of the relationship between CRs’ RMBPC (2.352, Std. Error  =  0.01, *P* = 0.02) and CGs’ mental health (Figure 4). However, from the results of mediation testing of MHPMC self-efficacy, no significant influence of MHPMC self-efficacy was found on MCS (β = .04, △R^2^ = .002, *P* = .546) after adjusting for RMBPC score. Although the β values of RMBPC were slightly increased from –.25 (*P* = .003) to –.24 (*P* = .009), no partial mediation effect of MHPMC self-efficacy was identified on the influences of BPSD on CGs’ mental health, from the result of Sobel test (1.727, Std. Error  =  0.01, *P* = 0.08).

## Discussion

The current study explored whether five domains of caregiver self-efficacy mediated the relationships between BPSD or four aspects of social support and CGs’ mental health. The results showed two domains of caregiver self-efficacy (gathering information and managing caregiving distress self-efficacy) were partial mediators of the relationship between positive social interaction support and CGs’ mental health. Managing caregiving distress self-efficacy was also a partial mediator of the relationship between CRs’ BPSD and CGs’ mental health.

The current study adds to the relevant literature [2], [3], [28]–[30] and examined the impact of each caregiver self-efficacy variable on CGs’ mental health. We found that CGs reported better mental health, when they had a stronger sense of self-efficacy with respect to gathering information and managing caregiving distress. The results were consistent with previous literature [8], [30], and can be fathomed by considering the nature of the two caregiver self-efficacy variables. Interestingly, we also identified a negative influence of managing routine care self-efficacy on CGs’ mental health. We inferred that CGs who were more confident in managing household, personal and medical care possibly engaged in more of these caregiving tasks, and subsequently increased their care involvement. Consequently, their mental health was jeopardized via increased stress both specific to the caregiving situation for a loved one and non-specifically, due to high demands on time and energy. Therefore, these findings implied that enhancing caregiver self-efficacy should be considered as an integral part in the interventions to improve CGs’ mental health, but the associations of specific domains of caregiver self-efficacy with CGs’ mental health should also be taken into account in designing an effective intervention program.

The current study also confirms earlier findings [17], [31] that dementia-related impairments and CGs’ social support were the two important factors influencing CGs’ belief in their capacity to overcome caregiving challenges. The overall influence of the two factors was also significant for CGs’ mental health, a result also consistent to the related literature [8], [17], [32]. As most of the CRs were at a mild stage of dementia, this study did not found high levels of BPSD. However, the results of this study found BPSD directly impaired three domains of caregiver self-efficacy (responding to BPSD, managing routine care and caregiving distress) and mental health. The findings support the previous literature [9], [10], [33] that managing BPSD was the most challenging task in CGs’ daily caregiving activities. Moreover, the mediation effect of managing caregiving distress self-efficacy on the influence of BPSD on CGs’ mental health further emphasized the importance of enhancing CGs’ stress management self-efficacy when determining intervention strategies to improve their health-related outcomes.

In addition, our study found that the CGs did not receive high levels of social support, particularly emotional support obtained from friends and other family members. There is lack of adequate community-based data on formal and informal support to dementia CGs in China. A cross-cultural survey [34] reported that CGs living in urban areas of China obtained less informal social support than those in other developing countries. The results of our previous qualitative study [20] also identified this phenomenon. The present study further buttresses cognate literature. Our results also documented that each caregiver self-efficacy measure (gathering information, obtaining support, responding to BPSD, managing routine care and caregiving distress) was significantly influenced by specific types of social support. For example, gathering information self-efficacy was positively influenced by three types of social support (informational, affectionate support and positive social interaction support). Of the four types of social support, positive social interaction support positively influenced most domains of caregiver self-efficacy including gathering information, responding to BPSD and managing caregiving distress self-efficacy. Since the three domains of caregiver self-efficacy are associated with the scope of CGs’ social activities, effectiveness of symptom management and levels of CGs' subjective burden, the findings indicate the importance of social activities (particularly those involving positive social interaction) on caregiver self-efficacy. Moreover, our mediation testing demonstrated that positive social interaction support played a positive and crucial role in CGs’ mental health, directly and through CGs’ gathering information and managing caregiving distress self-efficacy influencing their mental health. Previous investigators [35], [36] have repeatedly noted the association of social interaction with improving CGs’ awareness of dementia and related care, reducing caregiver stress and improving CGs’ mental health. Providing information support to CGs has be regarded an indispensable strategy in the intervention literature. The findings of our study further implicated that to facilitate positive social interact could be an effective way to providing informational support to the CGs.

While the results presented here offer some clear guidance for practice, some limitations on the generality of the results should be noted. Those elements afford opportunities for future studies. The study did not find unique impacts of obtaining support and responding to BPSD self-efficacy on CGs’ mental health. Rather, we showed that the CGs’ perceiving less tangible support and caring for CRs with severer BPSD reported weaker senses of obtaining support self-efficacy and responding to BPSD self-efficacy, respectively. We inferred that the two situational factors may contribute to the insufficient influence of the two domains of caregiver self-efficacy on CGs’ mental health. The findings were inconsistent with some of the previous studies [2], [28]. Meanwhile, the current study did not show the roles of the other four caregiver self-efficacy variables on the influence of CRs’ impairments and CGs’ social supports on CGs’ mental health. These results indicate a need for further studies, particularly using longitudinal and multi-centered designs, to examine the variations in the relationships between the five caregiver self-efficacy variables and CGs’ mental health in the course of CRs’ illness. Potential ramifications may exist between self-report data and objective measures. Moreover, the limited sample size in this study also affected the exploration of intricate relationships among these variables.

In addition, the previous literature [6], [8], [32] has suggested that the domains of caregiver self-efficacy and level of CGs’ mental health were also influenced by CGs’ socio-demographic data. These were outside the scope of this study; however, these relationships will be reported in future studies. Overall, this study contributes to the literature applying the theoretical concept of self-efficacy to the increasingly important issue of dementia care, with all the psychological, sociological, medical, economic, and public policy implications of dementia care. Our findings further indicate that, in determining supportive programs for the target population in mainland China, some effective strategies can be considered to improve CGs’ mental health, including assisting with BPSD management and enhancing CGs’ gathering information and stress management self-efficacy through providing corresponding information and facilitating CGs’ positive social interaction. Therefore, the findings provide information to future research, particularly intervention studies, on dementia caregiving.
